# Preparedness for Zika virus testing in the World Health Organization Western Pacific Region

**DOI:** 10.5365/WPSAR.2016.7.1.007

**Published:** 2016-03-31

**Authors:** Raynal C Squires, Frank Konings, on behalf of the World Health Organization Regional Office for the Western Pacific Zika Incident Management Team

**Affiliations:** a Emerging Disease Surveillance and Response Unit, Division of Health Security and Emergencies, World Health Organization Regional Office for the Western Pacific, Manila, Philippines.

## Abstract

On 1 February 2016, the World Health Organization (WHO) declared that clusters of microcephaly cases and other neurological disorders occurring in Zika virus (ZIKV)-affected areas constituted a public health emergency of international concern. Increased surveillance of the virus, including the requirement for laboratory confirmation of infection, was recommended. The WHO Regional Office for the Western Pacific therefore initiated a rapid survey among national-level public health laboratories in 19 countries and areas to determine regional capacity for ZIKV detection. The survey indicated that 16/19 (84%) countries had capacity for molecular detection of ZIKV while others facilitated testing through referral. These results suggest that robust laboratory capacity is in place to support ZIKV surveillance in the Western Pacific Region.

Initially identified in a rhesus monkey from Uganda’s Zika forest in 1947 and subsequently isolated from humans in 1968 in Nigeria, (*1*) Zika virus (ZIKV) is a flavivirus transmitted by *Aedes* mosquitoes, the same vector transmitting other arboviruses of public health impact such as yellow fever virus, dengue virus (DENV) and chikungunya virus (CHIKV). (*2*) The first known ZIKV outbreak occurred in 2007 in Yap state of the Federated States of Micronesia (*1*) in the World Health Organization (WHO) Western Pacific Region followed by a 2013–2014 outbreak in French Polynesia with an estimated 32 000 cases. (*3*) The virus has gone on to cause outbreaks in multiple Pacific island countries and has spread throughout the Americas. (*1*) In November 2015, Brazil began reporting substantial increases in the number of children born with microcephaly in ZIKV-affected areas. (*4*) That evidence, coupled with reports of Guillain-Barré syndrome cases in other ZIKV outbreaks, particularly in French Polynesia, led WHO on 1 February 2016 to declare that the cluster of microcephaly cases and other neurological disorders constituted a public health emergency of international concern (PHEIC). (*5*) Among the recommendations from that meeting of the International Health Regulations (2005) Emergency Committee were that “surveillance for ZIKV infection should be enhanced, with the dissemination of standard case definitions and diagnostics to at-risk areas.” (*6*)

Laboratory testing is a critical component of surveillance for ZIKV infection due to co-circulation of DENV and CHIKV that cause similar symptoms. (*7*, *8*) To determine regional capacity for ZIKV detection, the WHO Regional Office for the Western Pacific initiated a voluntary, rapid survey among national-level public health laboratories in its countries and areas (areas are non-sovereign jurisdictions within a WHO region; (*9*) countries and areas are together referred to as “countries” in this article). The survey sought to assess preparedness for ZIKV testing in the context of co-circulating DENV and CHIKV. Questions primarily addressed in-country capacity for molecular and serological detection of the three arboviruses, additional laboratory capacities specific for ZIKV and testing-related services to other countries.

The 19-question, e-mail-based survey was administered between 2 and 23 February 2016, immediately following the PHEIC declaration. A total of 28 surveys to national-level laboratories likely to be tasked with ZIKV testing were distributed to 19 countries in the Region (omitting resource-limited countries with basic laboratory capacity known to rely on specimen referral). The survey was completed by 23 laboratories in 18 countries. For the country not responding, information from other sources such as recent peer-reviewed publications was used where possible to augment the data set and cover all 19 countries.

**Table 1** summarizes the main findings of the survey. Polymerase chain reaction (PCR)-based detection of ZIKV was in place for 16/19 (84.2%) countries. Of the remaining three, two were using specimen referral to neighbouring countries (similar to Pacific island countries without PCR capacity), while the other has been working closely with the WHO Regional Office for the Western Pacific to obtain materials and reagents to enable in-country testing. Of the 16 countries with PCR test capacity for ZIKV, 14 could additionally sequence the virus and isolate it in culture. Serological diagnosis of ZIKV infection by immunoglobulin M (IgM) and/or immunoglobulin G (IgG) detection was also surveyed in the 19 countries, with less than one third (6/19) reporting having this capacity. Twelve countries indicated that they were willing to accept international specimens to supplement the capacity in other countries or for confirmation testing (data not shown).

**Table 1:** Responses to an e-mail-based survey assessing national-level public health laboratory testing capacity for ZIKV and other priority arboviruses among 19 countries and areas* in the WHO Western Pacific Region, 2–23 February 2016

Category	Proportion of countries	%
*In-country molecular testing (PCR) available*		
PCR for DENV	17/19	89.5
PCR for CHIKV	17/19	89.5
PCR for ZIKV	16/19	84.2
*Related ZIKV techniques available*		
Sequencing of ZIKV	14/16	87.5
Isolation of ZIKV	14/16	87.5
*Differential diagnostic PCR algorithm^†^*		
Concurrent (US CDC algorithm (*7*))	9/15	60.0
Sequential (AMRO algorithm (*10*))	5/15	33.3
Case-by-case	1/15	6.7
*In-country serological testing available*		
IgM and/or IgG for DENV	17/19	89.5
IgM and/or IgG for CHIKV	16/19	84.2
IgM and/or IgG for ZIKV	6/19	31.6

* Countries and areas covered under the survey were: Australia, Brunei Darussalam, Cambodia, China, Fiji, French Polynesia (France), Hong Kong Special Administrative Region (China), Japan, the Lao People's Democratic Republic, Macau Special Administrative Region (China), Malaysia, Mongolia, New Caledonia (France), New Zealand, Papua New Guinea, the Philippines, the Republic of Korea, Singapore and Viet Nam.

^†^ Data unavailable from one country with PCR testing capacity for ZIKV.

AMRO, World Health Organization Regional Office for the Americas; CHIKV, chikungunya virus; DENV, dengue virus; PCR, polymerase chain reaction; US CDC, United States Centers for Disease Control and Prevention; ZIKV, Zika virus.Given the similarity of disease presentation, (*1*) co-circulation and increasing prevalence of infection, (*11*-*13*) differential diagnosis for DENV, CHIKV and ZIKV is crucial. Molecular detection of DENV and CHIKV was in place in 17/19 (89.5%) countries, and a similarly large majority could perform serological diagnosis of DENV (17/19, 89.5%) and CHIKV (16/19, 84.2%) infection by IgM and/or IgG detection. The algorithm followed for differential diagnosis should take into consideration the endemic circulation of DENV, CHIKV and ZIKV. (*8*) Among 15 countries detailing their algorithm, 9 (60%) indicated they tested suspected samples for all three arboviruses concurrently, similar to the algorithm recommended by the United States Centers for Disease Control and Prevention; (*7*) 5/15 (33.3%) attempted to rule out each virus sequentially as outlined in the WHO Regional Office for the Americas guidance. (*10*) The remaining country indicated that the epidemiological circumstances of each case drove the specific algorithm followed.

This survey, conducted immediately following the WHO declaration of a PHEIC surrounding clusters of microcephaly and neurological disorders in the context of ZIKV infection, suggests that robust coverage for molecular detection of priority arboviruses is in place in the Region. Molecular detection by PCR is the critical differential diagnostic tool in this public health event as serology is problematic due to antibody cross-reactivity in regions with multiple circulating flaviviruses and/or use of vaccines against those viruses (for example, Japanese encephalitis virus). (*7*, *8*) Other methodologies, such as the plaque reduction neutralization test (PRNT), exist for the specific serological discrimination among the flaviviruses but require significant technical expertise for accurate execution and would not be practical for large-scale surveillance. Only three of the countries responding to the survey reported they could perform PRNT (data not shown).

As in our previous survey of regional PCR testing capacity for Middle East respiratory syndrome coronavirus, (*14*) most (13/16) of the national-level public health laboratories supporting PCR testing of ZIKV in their countries functioned as National Influenza Centres in the Global Influenza Surveillance and Response System, showing the versatility of this network. A similar proportion (12/16) participated in the external quality assessment (EQA) for DENV and CHIKV diagnostics, which has a substantial PCR-based component. This EQA, conducted by the WHO Regional Office for the Western Pacific (*15*, *16*) in 2013 and 2015, showed robust proficiency for diagnosis of these viruses in the Region.

While the Region seems prepared overall for testing of ZIKV, it is important to continue strengthening the apparatus for detection particularly through ensuring testing proficiency by EQA participation and enhancing referral mechanisms and International Air Transport Association certification where needed. It should be noted that while the EQA for DENV and CHIKV diagnostics gives confidence about regional testing proficiency for those arboviruses, (*16*) proficiency of ZIKV diagnostics remains untested. Note also that by omitting countries known to rely mainly on specimen referral, the study’s geographic coverage only included countries of the Asian subregion and larger countries or referral hubs of the Pacific subregion such as Australia and French Polynesia.

The laboratory plays an important role in improving our understanding of ZIKV epidemiology. While this survey reveals a broad availability of molecular diagnostics to support surveillance of ZIKV in the Western Pacific Region, further key roles remain for laboratories in helping to unravel the pathogenicity of the virus and its potential causal role in the observed cases of microcephaly and other neurological disorders.
